# Novel technique for generating macrophage foam cells for in vitro reverse cholesterol transport studies[S]

**DOI:** 10.1194/jlr.M041327

**Published:** 2013-12

**Authors:** Bhaswati Sengupta, Chandrakala Aluganti Narasimhulu, Sampath Parthasarathy

**Affiliations:** Burnett School of Biomedical Sciences, College of Medicine, University of Central Florida, Orlando, FL 32827

**Keywords:** lysophosphatidylcholine, high density lipoprotein, NBD-cholesterol

## Abstract

Generation of foam cells, an essential step for reverse cholesterol transport studies, uses the technique of receptor-dependent macrophage loading with radiolabeled acetylated LDL. In this study, we used the ability of a biologically relevant detergent molecule, lysophosphatidylcholine (lyso-PtdCho), to form mixed micelles with cholesterol or cholesteryl ester (CE) to generate macrophage foam cells. Fluorescent or radiolabeled cholesterol/lyso-PtdCho mixed micelles were prepared and incubated with RAW 264.7 or mouse peritoneal macrophages. Results showed that such micelles were quite stable at 4°C and retained the solubilized cholesterol during one month of storage. Macrophages incubated with cholesterol or CE (unlabeled, fluorescently labeled, or radiolabeled)/lyso-PtdCho mixed micelles accumulated CE as documented by microscopy, lipid staining, labeled oleate incorporation, and by TLC. Such foam cells unloaded cholesterol when incubated with HDL but not with oxidized HDL. We propose that stable cholesterol or CE/lyso-PtdCho micelles would offer advantages over existing methods.

Increased plasma LDL, an important risk factor for atherosclerosis, leads to its subendothelial accumulation. In the intima, LDL undergoes several structural and physiological modifications, mediated by oxidation, denaturation, enzyme actions, or self-aggregation (1–5). Enhanced uptake of modified lipoproteins by macrophages involves at least two crucial scavenger receptors, namely, scavenger receptor class A type 1 (SR-A1) and cluster of differentiation 36 (CD36) (6, 7). In addition, fluid phase pinocytosis of LDL has been suggested to result in foam cell formation (8). Eventually, lesion progression and plaque rupture result in platelet aggregation and the formation of thrombus, which can lead to heart attack or stroke (9). Changes in lifestyle, diet, and physical exercise are known to cause regression of atherosclerosis (10).

Reverse cholesterol transport (RCT) is a mechanism through which cholesterol from peripheral tissues, especially from macrophage-rich plaques, is effluxed to apolipoprotein A-I/HDL, and then transferred to liver for disposal (11–13). It is currently believed that the cholesterol efflux capacity of HDL, and not the HDL cholesterol level, has positive correlation with antiatherogenicity (14). However, recent studies by Hazen and associates showed a remarkable association of RCT with increased risk for myocardial infarction, stroke, and death. Although RCT assay at the time of sample collection indicated cholesterol efflux activity from macrophages to apoB-depleted serum, as cholesterol acceptor, was inversely associated with risk of prevalent coronary artery diseases (CAD) (15), when subjects were followed for three years, a positive correlation between increased cholesterol efflux and increased CAD was observed. Regardless of the implications of RCT, it is obvious that RCT is an important phenomenon that requires not only further investigations but also more efficient methods to study. Cholesterol efflux from peripheral tissues is promoted by transporters ABCA1 and ABCG1 (16). RCT also involves uptake of free and esterified cholesterol by hepatic HDL receptor, scavenger receptor class B type 1 (SR-B1) (17).

HDL not only acts as an acceptor for plaque cholesterol (18) but also has been suggested to have several antiatherogenic functionalities, such as antioxidative, anti-inflammatory, and antithrombotic (19–21) properties. Oxidation of HDL renders it “dysfunctional” (22, 23), and this dysfunctionality has been suggested to be an important factor in the control of atherosclerosis. Previous studies showed that oxidation of apoA-I impairs the efflux of cholesterol and, thereby, RCT (24).

The currently available technique to study RCT in vitro involves the use of macrophage foam cells generated by incubation with radioactive-cholesterol-labeled ac-LDL. Macrophages incubated with radiolabeled ac-LDL are used as foam cells to study RCT with respect to HDL heterogeneity (25) and to investigate the role of ABCA1 in RCT (26, 27). In recent times, a novel approach has been taken for high-throughput cholesterol efflux studies. Macrophages incubated with radiolabeled free cholesterol were utilized to study cholesterol efflux in presence of serum from human subjects (15, 28). Fluorescently labeled cholesterol (NBD-cholesterol), which has the NBD probe attached to alkyl chain of cholesterol (29), has been used for several in vitro RCT studies. This method has been extended to study RCT in an in vivo model (30). Foam cells loaded with radiolabeled ac-LDL were injected intraperitoneally in animal models, and then distribution of labeled cholesterol in plasma, liver, and feces were followed and measured (12, 31). To understand and evaluate the functionality of HDL, a speedier and more reliable assay needs to be developed.

The current methods of labeling ac-LDL with radioactive or fluorescent tags suffer from major deficiencies. First, LDL has to be isolated from suitable donors, and ac-LDL needs to be prepared. These lipoproteins are inherently unstable and their shelf life is limited. Our previous studies have demonstrated that LDL undergoes oxidation even during the centrifugation involved in its preparation (1). Longer dialysis and other steps also promote oxidation, and such particles have to be stored with EDTA or other additives. Acetylation involves toxic chemicals, such as acetic anhydride, and additional dialysis steps are involved in its isolation. The incorporation of radioactive/fluorescent tags involves physical adsorption methods and another purification step to remove unincorporated tracers. In addition, there is variability in the extent of cholesterol incorporation into different preparations of ac-LDL that might offer inconsistent information regarding RCT when cholesterol efflux data from different patients are compared.

Thus, there is a need for an efficient method for incorporation of cholesterol into macrophages. Past studies from our laboratory demonstrated that a highly nonpolar molecule, β-carotene, could be very efficiently solubilized using lyso-PtdCho as a natural “detergent.” The resulting solution was optically clear and stable, and it provided an opportunity to enrich cellular β-carotene in a concentration-dependent manner (32). In this study, we use a similar technique to incorporate cholesterol or cholesteryl ester (CE) into macrophages to generate foam cells. lyso-PtdCho is very efficiently utilized by macrophages by different ways: *i*) an acyltransferase reaction yields phosphatidylcholine (PtdCho) from lyso-PtdCho; *ii*) lyso phospholipase cleaves the acyl ester bond and removes the detergent nature of lyso-PtdCho; and *iii*) intermolecular transesterification reaction creates a molecule of PtdCho and glycerophosphorylcholine (33, 34). In contrast, macrophages esterify cholesterol and accumulate CE efficiently (35). Thus, while lyso-PtdCho would be “de-toxified,” CE would accumulate. This process also could be considered biological, as oxidized LDL (ox-LDL) has been suggested to be involved in foam cell formation and contains substantial levels of lyso-PtdCho (36, 37).

NBD-cholesterol has been previously used in several studies related to uptake, metabolism, and HDL-mediated efflux in primary hepatocytes and fibroblasts (38, 39). In recent years NBD-cholesterol has become a significant tool to study cholesterol metabolism because it closely mimics the behavior of cholesterol regarding metabolism and intracellular trafficking (40). In the present study NBD-cholesterol along with a ^3^H-cholesterol has been used as the analog for cholesterol to demonstrate lyso-PtdCho-mediated cholesterol loading, foam cell development, and HDL-induced cholesterol efflux.

## MATERIALS AND METHODS

### Reagents

RPMI 1640, advanced DMEM (ADMEM), FBS, sodium pyruvate, L-glutamine, penicillin-streptomycin (PS), 1× PBS, NBD-cholesterol were bought from Invitrogen Life Technologies (Carlsbad, CA). Cholesterol, lyso-PtdCho from egg yolk, oleic acid, 0.9% saline, fucoidan, polyinosinic acid, snake venom phospholipase A_2_, 4-dimethylaminopyridine, and silica gel matrix for TLC separation were purchased from Sigma-Aldrich (St. Louis, MO). Chloroform, methyl alcohol, hexane, diethyl ether, acetic acid isopropyl alcohol, and ethyl alcohol were purchased from VWR international (Randor, PA). [1, 2-^3^H (N)] cholesterol, [1-^14^C] oleic acid, and [methyl-^14^C] choline chloride were purchased from American Radiolabeled Chemicals (St. Louis, MO). Oil Red O was purchased from Fisher Scientific (Hampton, NH) and oleic anhydride from Santa Cruz Biotechnology (Dallas, TX).

### Cell culture

RAW 264.7 macrophages were cultured in RPMI 1640 medium containing 10% FBS, 1% PS, 1% sodium pyruvate, and 1% L-glutamine. Cells were seeded at 1.2 × 10^6^/ml density and incubated at 37°C in a 5% CO_2_ incubator to reach 65–70% confluence overnight. For the experiments involving foam cell development, RPMI 1640 with 0.1% lipoprotein-deficient serum (LPDS) was used as the incubation medium. Swiss Webster mice were purchased from Charles River (Wilmington, MA) and used to collect peritoneal macrophages. Macrophages from peritoneal cavity of ten-week-old Swiss Webster mice were isolated by peritoneal lavage using 5 ml cold 0.9% saline, followed by centrifugation. Cells were cultured under the same conditions as used for RAW macrophages. HepG2 cells were cultured in ADMEM containing 10% FBS, 1% PS, and 1% L-glutamine.

### Solubilization of cholesterol

Stock solutions of 5 mM cholesterol and 2 mM NBD-cholesterol were prepared in chloroform. Lyso-PtdCho stock solution (20 mM) was prepared in chloroform:methanol (1:1). For a typical reaction, 3 µmol of cholesterol (600 µl from the stock), 10 nmol of NBD-cholesterol (5 µl from the stock), and 3 µmol of lyso-PtdCho (150 µl from the stock) were mixed together in a test tube, followed by drying under nitrogen. Sterile PBS (1 ml) was added to the dried content and the mixture was vigorously vortexed for 1–2 min. The aqueous solution was filtered through 0.22 µm filter. The entire procedure was done under semidarkness.

In studies with radioactive cholesterol, NBD-cholesterol was substituted with ^3^H-cholesterol to give a final specific radioactivity of 5,000 DPM/nmol of cholesterol.

### Synthesis of fluorescent CE

Two milligrams of cholesterol, 0.2 mg NBD-cholesterol, and 25 mg oleic anhydride were mixed and dried in a glass tube. Then 4-dimethylaminopyridine was added to the reaction mixture as a catalyst for esterification (41), and the whole reaction mixture was heated at 80°C for 30 min until the liquid became bright yellow. The presence of NBD-CE was verified by TLC, and later NBD-CE was separated from the reaction mixture by column chromatography. Purity of NBD-CE was checked again by TLC.

### Isolation and modifications of lipoproteins

Following Institutional Review Board approval, blood was collected in heparinized tubes from consenting healthy donors and stored on ice. Blood was centrifuged at 3,000 rpm for 20 min, and plasma was separated. Lipoproteins were isolated from normal plasma by sequential ultracentrifugation using a Beckman TL-100 tabletop ultracentrifuge (Beckman, Palo Alto, CA) (42). The isolated lipoproteins were dialyzed against 0.3 mM EDTA in 1× PBS (pH 7.4) overnight and subsequently filter sterilized. The amount of protein was estimated using the Folin Lowry method. Lipoprotein sample was subjected to oxidation immediately after dialysis. Oxidation of lipoproteins was performed using either 5 µM copper (Cu) or myeloperoxidase (MPO) as described previously (43, 44). LDL was acetylated using acetic anhydride (4). Acetylated LDL (ac-LDL) was dialyzed overnight and filter sterilized using 0.2 μm syringe filter. Isolated and modified lipoproteins were stored at 4°C and used within one week of preparation.

Tagging ac-LDL with ^3^H-cholesterol was done as described previously by Kritharides et al. (42, 43). ac-LDL and ^3^H-cholesterol in ethanol were coincubated with RPMI containing 1% LPDS for 24 h at 37°C. This stock reaction mixture was diluted to obtain a working solution that contained 10 µg protein/ml of ac-LDL and 0.25 µCi/ml ^3^H-cholesterol in RPMI with 0.1% LPDS (45, 46).

### Cellular uptake of cholesterol

RAW 264.7 macrophages were seeded in 12-well plates at 1.2 × 10^6^ cells/ml concentrations to reach 65–70% confluence overnight. Cells were washed with warm sterile PBS once, followed by 4 h of incubation with 0.1% LPDS containing RPMI 1640. After 4 h, these cells were incubated with 40 µl of cholesterol or CE/lyso-PtdCho mixed micelle filtrate per ml of medium [equivalent of 120 µM concentrations of lyso-PtdCho and cholesterol (or CE) each]. For each experiment involving cholesterol (unlabeled and NBD or ^3^H-cholesterol)/lyso-PtdCho mixed micelles, 40 µM oleic acid was added to the cells during the incubation. Freshly isolated peritoneal macrophages were seeded in 24-well plates at 2.5 × 10^6^ cells/ml concentrations and used in the same way to demonstrate cholesterol uptake via cholesterol/lyso-PtdCho mixed micelles. After overnight incubation, cells were washed with warm PBS twice, and images of live cells were taken under fluorescence microscope (Axio imager, Carl Zeiss AG, Oberkochen, Germany) or cells were fixed with 10% formalin and stained with Oil Red O.

### Detection of foam cells

To detect the presence of fluorescent NBD-cholesterol within the cells, images of live cells were taken with AxioCam MRm (Axio imager, Carl Zeiss AG, Oberkochen, Germany). RAW 264.7 macrophages were then lysed by incubating with 1 ml of methanol at 37°C for 15 min. Cell lysates were centrifuged at 3,000 rpm for 10 min. Supernatant (100 µl) was used to measure fluorescence intensity present within the cells. Fluorescence plate reader (Envision 2014 Multilabel Plate Reader, PerkinElmer, Waltham, MA) was used to measure fluorescence intensity at emission spectra of 535 nm upon excitation at 475 nm.

Macrophages were also fixed to stain for CE droplets. After washing with PBS, cells were fixed with 10% formalin, made permeable with 60% isopropanol, and stained with Oil Red O. Images of foam cells were taken under 10× and 40× objectives in the light microscope with the Leica DFC295 camera (Leica Camera AG, Solms, Germany). Oil Red O-stained lipid droplets were further quantified by elution of the stain, followed by absorption measurement with a spectrophotometer. Stained wells were rinsed with 60% isopropanol very briefly to remove any residual Oil Red O and then dried completely. Stains were eluted with 1 ml isopropanol for 10 min at room temperature. After thorough pipetting to ensure complete elution, 100 µl aliquots were taken in triplicates in a 96-well plate, and absorbance was measured at 500 nm (47).

### Quantification of cholesterol incorporated in total extracted lipid and in CE

Macrophages were incubated with ^3^H-cholesterol labeled ac-LDL or cholesterol (unlabeled and ^3^H-cholesterol)/lyso-PtdCho mixed micelles for 18 h at 37°C. Lipid extraction was done using the method of Bligh and Dyer (48). Lipids were dissolved in 50 µl of chloroform, and then 5 µl of this was utilized directly to measure ^3^H-cholesterol incorporation. This procedure was done in triplicate and the rest of the reconstituted lipid (∼35 µl) was used for TLC analysis. Lipids were separated by TLC on silica gel matrix using hexane/diethyl ether/acetic acid (30:6:0.5; v/v/v) solvent system (49) and visualized with iodine vapor. Spots of CE from samples incubated with ^3^H-cholesterol labeled ac-LDL or cholesterol (unlabeled and ^3^H-cholesterol)/lyso-PtdCho mixed micelles were compared with CE standard and analyzed for ^3^H-cholesterol incorporation by liquid scintillation and luminescence counters (MicroBeta2 Plate Counter, PerkinElmer, Waltham, MA). Count per minute (CPM) values obtained for the incorporation of ^3^H-cholesterol in total extracted lipid or CE fraction of lipid was converted into nanomoles of cholesterol and represented subsequently.

### Incorporation of ^14^C-oleic acid in CE within foam cells

To demonstrate the conversion of cholesterol into CE while using cholesterol/lyso-PtdCho mixed micelles, ^14^C-oleic acid was introduced during incubation of cells with cholesterol/lyso-PtdCho mixed micelles. A stock solution of 100 mM oleic acid containing 1 µCi ^14^C-oleic acid was prepared. RAW 264.7 macrophages were incubated with either cholesterol/lyso-PtdCho or CE/lyso-PtdCho mixed micelles in presence of 40 µM oleic acid/ml of medium for 18 h. After overnight incubation, cells were washed with warm PBS twice and lipid extraction was done with methanol/chloroform (2:1) (48). Chloroform-lipid phase was separated, the solvent was evaporated under nitrogen, and the lipids were reconstituted with chloroform. Lipids were separated and CE was identified as described previously. Spots of CE from samples incubated with cholesterol/lyso-PtdCho or CE/lyso-PtdCho mixed micelles were compared with CE standard and analyzed for ^14^C-oleic acid incorporation by autoradiography (Cyclone Plus Phosphor Imager, PerkinElmer, Waltham, MA) followed by liquid scintillation and luminescence counting.

### Measurement of lactate dehydrogenase activity

Lactate dehydrogenase (LDH) cytotoxicity assay kit (Catalog number 10008882) was purchased from Cayman Chemical (Ann Arbor, MI), and the assay was performed following the protocol supplied with the kit. In brief, RAW 264.7 macrophages were seeded at from 10^5^ to 10^6^ density in 96-well plates. After treating the cells for 18 h, 100 µl of medium was collected from each of the well and NAD^+^ (provided with the kit) was added to the medium. Enzymatic activity of LDH oxidized lactate into pyruvate and converted NAD^+^ into NADPH and H^+^. Finally, a colorimetric reaction between NADPH and H^+^ and diaphorase (provided with the kit) produced dark-pink formazan. Formation of formazan was measured at 490–520 nm wavelength using a plate reader (Benchmark Plus Microplate Spectrophotometer System, Bio-Rad, Hercules, CA), and LDH activity per ml of medium was calculated using standard curve. All the treatments were performed at least in triplicate and repeated four times.

### Hemolysis of human RBC

Human blood collected in a heparinized tube was centrifuged at 3,000 rpm for 20 min, and RBC was collected from the pellet and washed. RBC was diluted with 0.9% saline (1:20; v/v) and hemolysis in presence of water was performed to determine the optical density value equivalent to 1 and to determine the optimum volume of diluted RBC required for the rest of the experiment. Then 75 µl of diluted RBC was taken in eight glass tubes and 0, 10, 25, 50, 100, 150, 200, and 250 µM of lyso-PtdCho micelles, cholesterol/lyso-PtdCho, and CE/lyso-PtdCho mixed micelles were added to each tube. The volume was made up to 1 ml by adding PBS, and the reaction mixtures were incubated at room temperature for 15 min. Then the reaction mixtures were centrifuged at 2,000 rpm for 15 min. Without disturbing the cell pellet, 100 µl of supernatant from each glass tube were taken in triplicate in a 96-well plate and absorbance was measured at 540 nm.

### Synthesis of ^14^C-lyso-PtdCho and quantification of lyso-PtdCho metabolism by macrophages

Synthesis of ^14^C-lyso-PtdCho was done from ^14^C-choline chloride. HepG2 cells were incubated with 5 µCi of ^14^C-choline chloride in presence of total growth medium ADMEM that contains 4 µg/ml choline chloride. After 72 h of incubation, lipids were extracted and were separated by TLC using chloroform/methanol/water (65: 25: 3.5; v/v/v) as the solvent system. PtdCho band was extracted with chloroform: methanol (1:1) followed by reconstitution of dried lipid content with 0.8 ml of PBS. This reconstituted PtdCho was subjected to enzymatic conversion into lyso-PtdCho by adding 25 µl snake venom PLA_2_ (0.5 units) and 80 µl of 10 mM CaCl_2_. The reaction was set at 37°C for 4 h and the reaction mixture was used to run TLC using the previous solvent system with lyso-PtdCho and PtdCho as standards. lyso-PtdCho band from the TLC plate was extracted and was used for further experiments.

^14^C-lyso-PtdCho was mixed with 3 µmoles unlabeled lyso-PtdCho and 3 µmoles cholesterol from the previously described stock solutions. The reaction mixture was dried under nitrogen and reconstituted with 1 ml PBS by vigorous vortexing. After filtering, 5 µl of the micelles were used to measure radioactivity count. Cholesterol/lyso-PtdCho (unlabeled and ^14^C labeled) mixed micelles containing at least 5,000 CPM/ml activity (∼25 µl/ml of medium) were used to incubate RAW 264.7 macrophages with for 18 h at 37°C. After overnight incubation, lipid extraction was done by Bligh and Dyer method and reconstituted lipid was used to run TLC with PtdCho and lyso-PtdCho standards using chloroform/methanol/water (65: 25: 3.5; v/v/v) solvent system. Spots corresponding to PtdCho and lyso-PtdCho standards were isolated from the TLC plate, and metabolic fate of ^14^C-lyso-PtdCho was determined by liquid scintillation and luminescence counters.

### Quantification of cellular cholesterol efflux

RAW 264.7 macrophages and mouse peritoneal macrophages incubated overnight with cholesterol/lyso-PtdCho mixed micelles were washed with PBS twice, and fresh medium was added. Cells were then incubated with 0–200 µg/ml of native HDL or 25 and 50 µg/ml of ox-HDL (MPO or Cu mediated) for 4 h, and medium was collected to measure fluorescence intensity. After removal of the medium, RAW 264.7 macrophages were washed with PBS, and images under fluorescence microscope were taken. To measure fluorescence intensity within the cells, cell lysates were prepared as described before, or cells were fixed and stained with Oil Red O to observe CE accumulation.

To study cholesterol efflux, foam cells were also developed using ^3^H-cholesterol (and unlabeled cholesterol)/lyso-PtdCho mixed micelles. Efflux of ^3^H-cholesterol in medium upon incubation with HDL was measured by liquid scintillation and luminescence counters.

### Statistical analysis

Each experiment was performed more than three times, and each experimental condition was set up in triplicate. All values were presented as mean ± SD. To determine the difference between two groups, unpaired two-tailed Student *t*-test was applied. ANOVA (ANOVA) was applied with Dunnett's or Bonferroni's correction for multiple comparisons using GraphPad Prism 5.0 software (San Diego, CA). The minimum level of significance in all tests was *P* < 0.05.

## RESULTS

### Solubilization of cholesterol in aqueous solution in presence of lyso-PtdCho

**Fig. 1.**
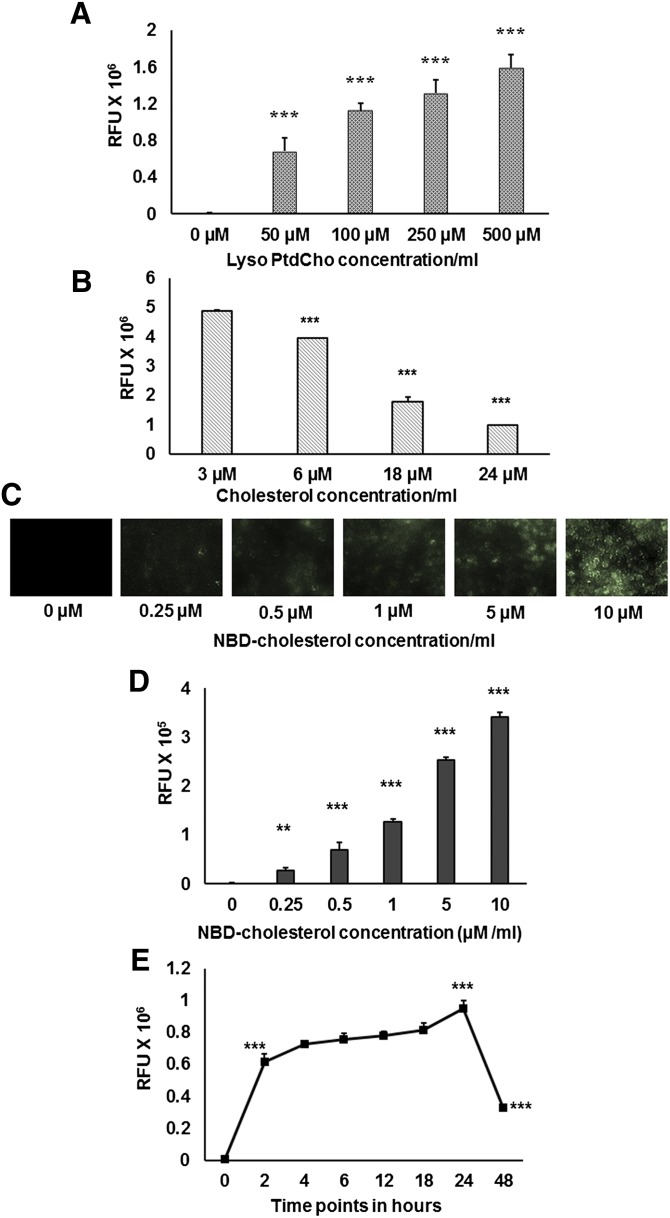
Solubilization of NBD-cholesterol/lyso-PtdCho micelles: Cholesterol (unlabeled) and NBD-cholesterol were mixed together and solubilized in presence of lyso-PtdCho to form mixed micelles. (A) Solubilization of cholesterol and NBD-cholesterol in presence of increasing concentrations of lyso-PtdCho. (B) Solubilization of NBD-cholesterol is inhibited by cholesterol in a concentration-dependent manner. (C) Concentration-dependent increased uptake of fluorescent cholesterol by RAW 264.7 macrophages. Cells were treated with mixed micelles containing 0–10 µM NBD-cholesterol; after 18 h, cells were visualized under fluorescence microscope (40× objectives). (D) Cells treated as above were lysed using methanol, and fluorescence intensity was measured using fluorescence plate reader. (E) Cells were treated with cholesterol and NBD-cholesterol/lyso-PtdCho mixed micelles for 0–48 h, and then fluorescence intensity in the cell lysate was measured. Values are expressed as mean ± SD (n ≥ 3). **P < 0.01, ***P < 0.005 (one-way ANOVA with Dunnett's multiple comparison test).

Cholesterol was solubilized in PBS by mixing cholesterol and lyso-PtdCho together. To determine if lyso-PtdCho could increase the solubility of cholesterol in PBS, constant amounts of cholesterol, NBD-cholesterol, and an increasing amount of lyso-PtdCho were taken in five glass tubes. After drying, aqueous solution was made with 1 ml PBS as described in Materials and Methods. Prepared mixed micelles contained 1 mM cholesterol (200 µl from 5 mM stock) and 5 µM NBD-cholesterol (2.5 µl from 2 mM stock) and lyso-PtdCho ranging from 0–500 µM (0, 2.5, 5, 12.5, and 25 µl from 20 mM stock). Fluorescence intensity was determined in the clear filtrate. Fluorescence intensity of the aqueous solution containing only cholesterol and fluorescent cholesterol (but no lyso-PtdCho) was at the background level and increased in the filtrates containing increasing amounts of lyso-PtdCho (**Fig. 1A**).

To determine whether cholesterol could reduce the solubility of NBD-cholesterol in PBS in a concentration-dependent manner, increasing concentrations of cholesterol ranging 3–24 µM (0.6, 1.2, 3.6, and 4.8 µl from 5 mM stock) were taken in four glass tubes. Equimolar concentration of lyso-PtdCho ranging 3–24 µM (0.15, 0.3, 0.9, and 1.2 µl from 20 mM stock) was added to the cholesterol, and a constant concentration of 5 µM NBD-cholesterol was taken. Mixed micelles in 1 ml of PBS were made as described above. The fluorescence intensity of the aqueous solution gradually decreased as the concentration of unlabeled cholesterol used to make mixed micelles increased (Fig. 1B).

### Accumulation of fluorescent cholesterol in macrophages

RAW 264.7 macrophages were incubated with mixed micelles containing increasing concentrations (0–10 µM) of NBD-cholesterol for 18 h. Then the cells were washed with PBS twice and visualized under fluorescence microscope (Fig. 1C). Macrophages showed concentration-dependent increased accumulation of NBD-cholesterol in the cytoplasm, which was further confirmed by measuring the fluorescence intensity in the corresponding cell lysates (Fig. 1D). Fluorescence intensity in cells incubated with 0.25, 0.5,1, 5, and 10 µM of NBD-cholesterol were observed to be several-fold higher than the background level.

### Mixed micelles-mediated cholesterol uptake is a quick process

RAW 264.7 macrophages were incubated with cholesterol (unlabeled and NBD-cholesterol)/lyso-PtdCho mixed micelles for 0, 2, 4, 6, 12, 18, 24, and 48 h. Cells for each treatment condition were incubated with 90 µM cholesterol, 300 nM NBD-cholesterol, and 90 µM lyso-PtdCho (15 µl of mixed micelles/500 µl of medium). At the end of each time point, cells were observed under microscope (data not shown) and were lysed to quantify fluorescence intensity in the cell lysates. It was observed that within 2 h, the fluorescence intensity of the cells treated with mixed micelles increased significantly from the background level. Furthermore, fluorescence intensity in cell lysates after treatment for 4 h increased slightly from 2 h and almost reached a plateau between 4 h and 18 h. However, after a steady increase in fluorescence intensity at 24 h, there was a sharp decrease in fluorescence intensity level at the 48 h time point even though it was still several-fold higher than the background level (Fig. 1E).

### Mixed micelles of cholesterol in aqueous solution are stable

**Fig. 2.**
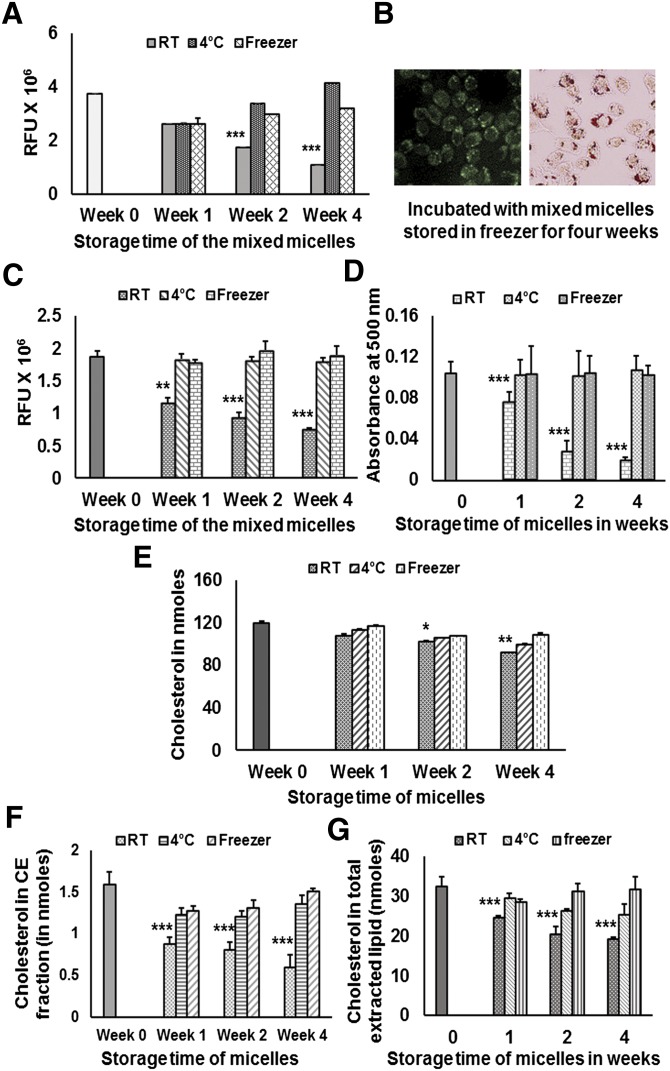
Stability of NBD-cholesterol/lyso-PtdCho micelles. (A) Cholesterol and NBD-cholesterol/lyso-PtdCho mixed micelles were stored at room temperature, 4°C, and −20°C for weeks 0–4, and the fluorescence intensity of the micellerized NBD-cholesterol solution stored at different temperatures for up to four weeks was measured. (B) Foam cells containing NBD-cholesterol were developed by incubating macrophages with mixed micelles stored at −20°C for four weeks. (C) Cells were treated with fluorescently labeled mixed micelles stored at room temperature, 4°C, and −20°C for weeks 0–4, and then fluorescence intensity in the cell lysates was measured. (D) Absorbance value of Oil Red O stain eluted after staining the cells as treated above. (E) Cholesterol, ^3^H-cholesterol/lyso-PtdCho mixed micelles were stored at room temperature, 4°C, and −20°C for weeks 0–4, and then radioactivity of the micellerized cholesterol solution stored at different temperatures for up to four weeks was measured (CPM values converted into nmol of cholesterol). (F) cells were treated with ^3^H-cholesterol-labeled mixed micelles stored at room temperature, 4°C, and −20°C for weeks 0–4, and then incorporation of cholesterol in the CE fraction was measured. (G) Incorporation of cholesterol in the total extracted lipid after the treatment of cells was measured as mentioned above. Values are expressed as mean ± SD (n ≥ 3). *P < 0.05, **P < 0.01, ***P < 0.005 (one-way ANOVA with Dunnett's multiple comparison test). Significant differences in values for different storage temperatures and time points are compared with week 0 value.

Mixed micelles of cholesterol, NBD-cholesterol, and lyso-PtdCho in PBS was prepared as described before, and aliquots of the filtrate were stored at room temperature, 4°C, and −20°C. Fluorescence intensity of the filtrate from each storing condition was determined at week 0, week 1, week 2, and week 4 from the storage time. A mixed micelle solution from each temperature was filtered again immediately before its use to measure fluorescence intensity or to treat the cells. We observed that the fluorescence intensity of the solution stored at room temperature started deteriorating rapidly, while the fluorescence intensity of the solutions stored at 4°C and −20°C was fairly stable for up to four weeks (**Fig. 2A**). Furthermore, the mixed micelles stored at −20°C for four weeks were used to develop macrophage foam cells (Fig. 2B). Fluorescent cholesterol accumulation in the macrophages was documented by taking images under fluorescence microscope, and droplets of CE were identified by Oil Red O staining.

Fluorescent cholesterol accumulation was further quantified by measuring fluorescence intensity in the cell lysates (Fig. 2C), and the accumulated CE droplets were quantified by eluting the Oil Red O stain (Fig. 2D). Measurement of fluorescence intensity and elution of Oil Red O stain confirmed the previous observations regarding the stability of mixed micelles at different temperatures. Cells treated with micelles stored at 4°C and −20°C showed almost no change in NBD-cholesterol or CE droplet accumulation from the starting time point (week 0). However, there was a sharp decrease in the accumulation of NBD-cholesterol and CE droplets in cells treated with micelles stored at room temperature.

To substantiate the observations regarding the stability of mixed micelles, additional experiments were performed. Mixed micelles were prepared using cholesterol (unlabeled and ^3^H-cholesterol) and lyso-PtdCho, and filtered micelles were stored as described in the previous section. Measurements of radioactivity in the micelles stored at room temperature, 4°C, and −20°C were done at week 0, week1, week 2, and week 4. Micelles stored under these conditions were further used to incubate RAW 264.7 macrophages for 18 h. Each time before using, the micelles stored at different temperatures were filtered through 0.22 µm filter. Stability of the ^3^H-cholesterol-containing micelles (Fig. 2E) mimicked the results of micelles containing NBD-cholesterol. More interestingly, cells treated with 40 µl (per milliliter of medium) of ^3^H-cholesterol-containing micelles stored at room temperature showed steadily decreasing incorporation of cholesterol in total extracted lipid (Fig. 2G) as well as in CE fraction (Fig. 2F). Incorporation of cholesterol in total extracted lipid (Fig. 2G) and in CE fraction (Fig. 2F) remained almost the same as that of the freshly prepared micelles when cells were treated with micelles stored at 4°C and −20°C even after four weeks (TLC plate, supplementary Fig. I).

### Mixed micelles mediated cholesterol uptake does not cause cell cytotoxicity

**Fig. 3.**
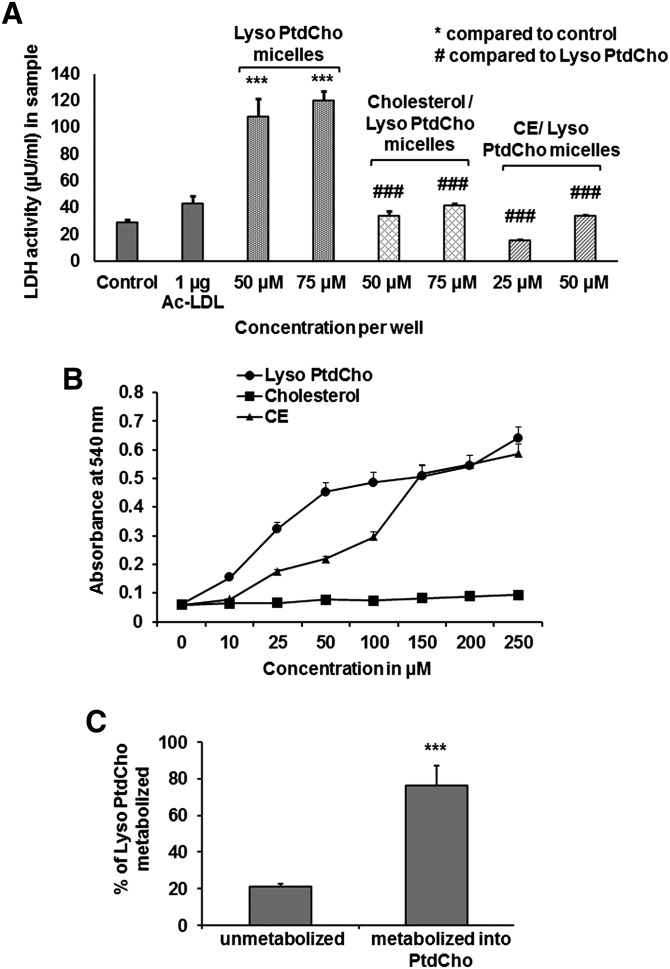
Foam cells developed using cholesterol or CE (unlabeled)/lyso-PtdCho mixed micelles without inducing cytotoxic effects. (A) Lactate dehydrogenase secreted in the medium was used to quantify LDH activity (µU/ml) in samples treated under different conditions: no treatment, 1 µg ac-LDL, 50 and 75 µM lyso-PtdCho micelles, or cholesterol/lyso-PtdCho micelles, 25 and 50 µM CE/lyso-PtdCho micelles per well. *P* values for samples treated with lyso-PtdCho alone were calculated against control (no treatment). For samples treated with cholesterol or CE/lyso-PtdCho, mixed micelles were calculated against the samples treated with lyso-PtdCho alone. Values are expressed as mean ± SD (n ≥ 3). *** P < 0.005, ^###^*P* < 0.005 (one-way ANOVA with Bonferroni's multiple comparison test). (B) Hemolysis of human RBC was demonstrated by adding increasing concentrations of lyso-PtdCho micelles, cholesterol/lyso-PtdCho, or CE/lyso-PtdCho mixed micelles to constant concentration of RBC, and then the optical density of the reaction mixture was measured. (C) Metabolism of ^14^C-lyso-PtdCho used as the component of cholesterol/lyso-PtdCho micelles is shown. Values are expressed as mean ± SD (n ≥ 3). ***P < 0.005 (two-tailed Student *t*-test).

The amount of lactate dehydrogenase secreted into the medium during the incubation of cells with cholesterol (or CE)/lyso-PtdCho mixed micelles was measured to rule out the cytotoxic effect of the mixed micelles (**Fig. 3A**). Mixed micelles contained 3 mM lyso-PtdCho alone, 3 mM cholesterol, or CE with equimolar lyso-PtdCho. Macrophages were seeded in 96-well plates in 1 × 10^5^/well concentration, and after 24 h, were washed with PBS twice and incubated with 120 µl of RPMI containing 0.1% LPDS for 4 h. Cell were then incubated with 1 µg ac-LDL per well (Fig. 3A, bar 2); 50 µM (2 µl from 3 mM lyso-PtdCho containing mixed micelles) and 75 µM (3 µl) lyso-PtdCho micelles per well (Fig. 3A, bars 3 and 4); 50 µM (2 µl from 3 mM cholesterol/lyso-PtdCho containing mixed micelles) and 75 µM (3 µl) cholesterol/lyso-PtdCho mixed micelles per well (Fig. 3A, bars 5 and 6); and 25 µM (1 µl from 3 mM CE/lyso-PtdCho containing mixed micelles) and 50 µM (2 µl) CE/lyso-PtdCho mixed micelles per well (Fig. 3A, bars 7 and 8) for 18 h. All the treatments were done in triplicate, and at the end of 18 h, 100 µl of medium was collected from each well to measure LDH activity in the sample. In this colorimetric assay, pink formazan was measured. Cells incubated with lyso-PtdCho micelles produced a very dark-pink color, indicating very high cell lysis, whereas cells treated with cholesterol or CE/lyso-PtdCho mixed micelles produced a very pale pink color, as was observed in the cells without any treatment. Thus, cells treated with cholesterol or CE/lyso-PtdCho mixed micelles were devoid of toxicity.

### Cytotoxic effects of lyso-PtdCho are greatly reduced by the presence of cholesterol in mixed micelles

RBC diluted in saline were mixed with lyso-PtdCho micelles or cholesterol/lyso-PtdCho or CE/lyso-PtdCho mixed micelles containing 0, 10, 25, 50, 100, 150, 200, and 250 µM of lyso-PtdCho or cholesterol or CE. Cholesterol and CE containing mixed micelles also had equimolar concentrations of lyso-PtdCho. The reaction mixture containing lyso-PtdCho micelles showed almost immediate hemolysis, and optical density value of the supernatant of the reaction mixtures was several-fold higher when compared with the background level. Conversely, reaction mixture containing cholesterol/lyso-PtdCho mixed micelles showed almost no hemolysis, even at the end of 15 min incubation, and optical density value of the supernatant of the reaction mixtures was barely above the background level. Nevertheless, the hemolytic properties of CE/lyso-PtdCho mixed micelles were in between the other two micelles. At lower concentrations of 10–100 µM, CE/lyso-PtdCho did not cause very significant amount of hemolysis, even though the amount of hemolysis was higher than that induced by cholesterol/lyso-PtdCho mixed micelles. There was a sharp increase in the level of hemolysis in the reaction mixtures containing more than 100 µM CE/lyso-PtdCho mixed micelles. At 150–250 µM concentrations, levels of hemolysis caused by CE/lyso-PtdCho mixed micelles were almost the same as those of lyso-PtdCho micelles (Fig. 3B).

### lyso-PtdCho component of mixed micelles was metabolized into PtdCho

Cells treated with cholesterol/lyso-PtdCho (unlabeled and ^14^C-lyso-PtdCho) mixed micelles for 18 h demonstrated that more than 75% of lyso-PtdCho provided as the component of mixed micelles was metabolized into PtdCho (Fig. 3C). The metabolization of lyso-PtdCho to PtdCho was a very quick process, which took place within 2 h (data not shown) as demonstrated previously (50, 51).

### Foam cells are developed by incubating macrophages with cholesterol containing mixed micelles

**Fig. 4.**
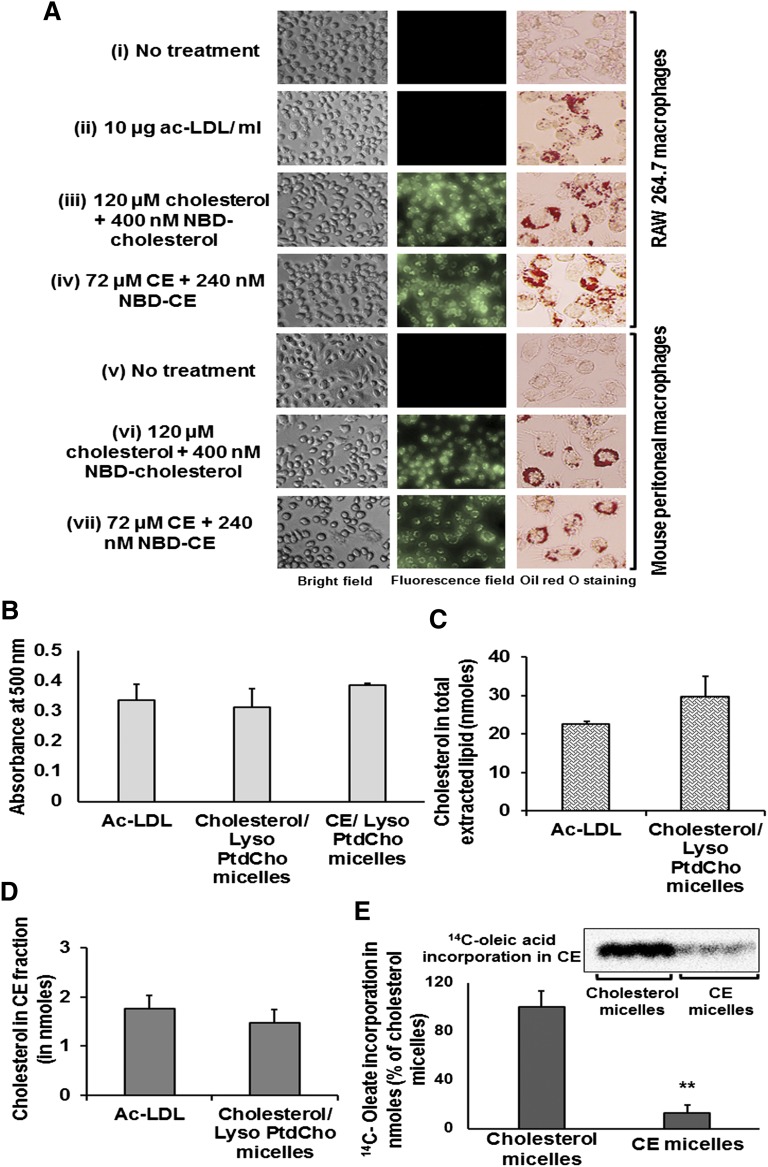
Foam cells developed using mixed micelles show cholesterol esterification. (A) RAW 264.7 (i–iv) and mouse peritoneal macrophages (v–vii) incubated with mixed micelles for 18 h show accumulation of NBD-cholesterol (iii, RAW 264.7 macrophages; vi, mouse peritoneal macrophages) and fluorescent CE (iv, RAW 264.7 macrophages; vii, mouse peritoneal macrophages) droplets under fluorescent microscope. CE droplet accumulation is demonstrated by Oil Red O staining. CE droplet accumulation was observed in cells treated with ac-LDL (ii, RAW 264.7 macrophages), cholesterol (iii, RAW 264.7 macrophages; vi, mouse peritoneal macrophages), or CE (iv, RAW 264.7 macrophages; vii, mouse peritoneal macrophages)/lyso-PtdCho mixed micelles. (B) Oil Red O stain from RAW 264.7 macrophages treated with ac-LDL and cholesterol/lyso-PtdCho or CE/lyso-PtdCho mixed micelles were eluted and absorbance was measured. (C) cells were treated with ^3^H-cholesterol-labeled ac-LDL or cholesterol, ^3^H-cholesterol/lyso-PtdCho mixed micelles and incorporation of cholesterol in total extracted lipid was measured. (D) Incorporation of cholesterol in CE fraction was measured following the treatment above. (E) Cells incubated with cholesterol or CE/lyso-PtdCho mixed micelles were supplied with 40 μM ^14^C-oleic acid/ml. Cells incubated with CE/lyso-PtdCho mixed micelles showed almost 10-fold less ^14^C-oleic acid incorporation than those treated with cholesterol/lyso-PtdCho mixed micelles. Insert shows radioautograph image of ^14^C-oleic acid incorporation in CE fraction of cells as treated above. Values are expressed as mean ± SD (n ≥ 3). ** *P* < 0.01 (two-tailed Student *t* -test).

RAW 264.7 macrophages and mouse peritoneal macrophages were incubated with mixed micelles (40 µl of cholesterol/lyso-PtdCho micelles/ml of medium or 24 μl of CE/lyso-PtdCho mixed micelles/ml of medium) for 18 h. Cholesterol/lyso-PtdCho mixed micelles contained 120 μM cholesterol (unlabeled), 400 nM NBD cholesterol, and 120 μM lyso-PtdCho (**Fig. 4A** iii,RAW macrophages, and vi, mouse peritoneal macrophages), whereas CE/lyso-PtdCho mixed micelles had 72 μM unlabeled CE, 240 nM fluorescently labeled CE, and 72 μM lyso-PtdCho (Fig. 4A iv, RAW macrophages, and vii, mouse peritoneal macrophages). ac-LDL (10 μg/ml, Fig. 4A ii) treated RAW macrophages were used as positive control for all the foam cell development studies. Macrophages incubated with cholesterol/lyso-PtdCho micelles were provided with 40 µM oleic acid to aid CE conversion and accumulation. When observed under the fluorescence microscope, cells treated with NBD-cholesterol containing mixed micelles showed fluorescent cholesterol accumulation within the cells. As expected negative control cells (Fig. 4A, i and v) and cells treated with ac-LDL (Fig. 4A, ii) did not yield any fluorescent images. To ensure the accumulation of fluorescent cholesterol was only within the cells, we lysed RAW 264.7 macrophages and measured the fluorescence intensity in the cell lysate. Fluorescence intensity in the cell lysates of negative control was comparable to that of the background level, whereas fluorescence intensity was much higher in the lysate of cells incubated with mixed micelles (data not shown).

CE droplet accumulation in RAW 264.7 macrophages was demonstrated by Oil Red O staining following the treatment with ac-LDL, cholesterol/lyso-PtdCho, and CE/lyso-PtdCho mixed micelles. Oil Red O stain was eluted after the images were taken. Eluted Oil Red O stain, which indirectly quantifies the amount of CE droplets present in the cells, was taken in 100 µl aliquots and absorption was measured at 500 nm. Optical density value of all the three samples matched very closely and ranged 0.3–0.4 (Fig. 4B).

### Quantity of CE accumulated in foam cells developed by ac-LDL mediated uptake or mixed micelles mediated uptake are similar

RAW 264.7 macrophages were treated with 10 µg/ml of ac-LDL labeled with ^3^H-cholesterol or with cholesterol (unlabeled and ^3^H-cholesterol)/lyso-PtdCho mixed micelles for 18 h. Quantification of cholesterol incorporation in total extracted lipid showed that foam cells developed by ac-LDL-mediated and mixed micelles-mediated uptake contained 22 nmol and 26 nmol of cholesterol (volume of total extracted lipid was 50 µl), respectively (Fig. 4C). Cholesterol incorporation in the CE fraction of extracted lipid was 1.5 nmol for ac-LDL-mediated foam cell development and 1.8 nmol for that of mixed micelles (Fig. 4D).

### Cholesterol is esterified into CE during micelle mediated foam cell formation

RAW 264.7 macrophages incubated with cholesterol/lyso-PtdCho mixed micelles were simultaneously treated with ^14^C-oleic acid to study the incorporation of radioactive oleate during the esterification of cholesterol into CE. Oleic acid (40 µM with 200 pCi radioactivity/ml of medium) was supplied during the incubation of cells with cholesterol or CE/lyso-PtdCho mixed micelles, and after overnight incubation, lipids were extracted using methanol/chloroform (2:1). TLC separation of the samples from both types of micelle treatments showed distinct spots of CE when compared with the standard CE spot, and these spots were further analyzed for radioactivity. Before exposing the TLC plate to iodine vapor, a radioautograph image of the TLC plate was taken, and the image clearly showed much higher oleate incorporation in cells incubated with cholesterol/lyso-PtdCho micelles (Fig. 4E, insert). This observation was further confirmed when analysis of oleate incorporation in CE showed that cells incubated with CE/lyso-PtdCho micelles had a 10-fold percentage decrease in ^14^C-oleic acid incorporation in CE compared with cells treated with cholesterol/lyso-PtdCho micelles (Fig. 4E).

### Uptake of cholesterol micelles is distinct from that of ac-LDL

**Fig. 5.**
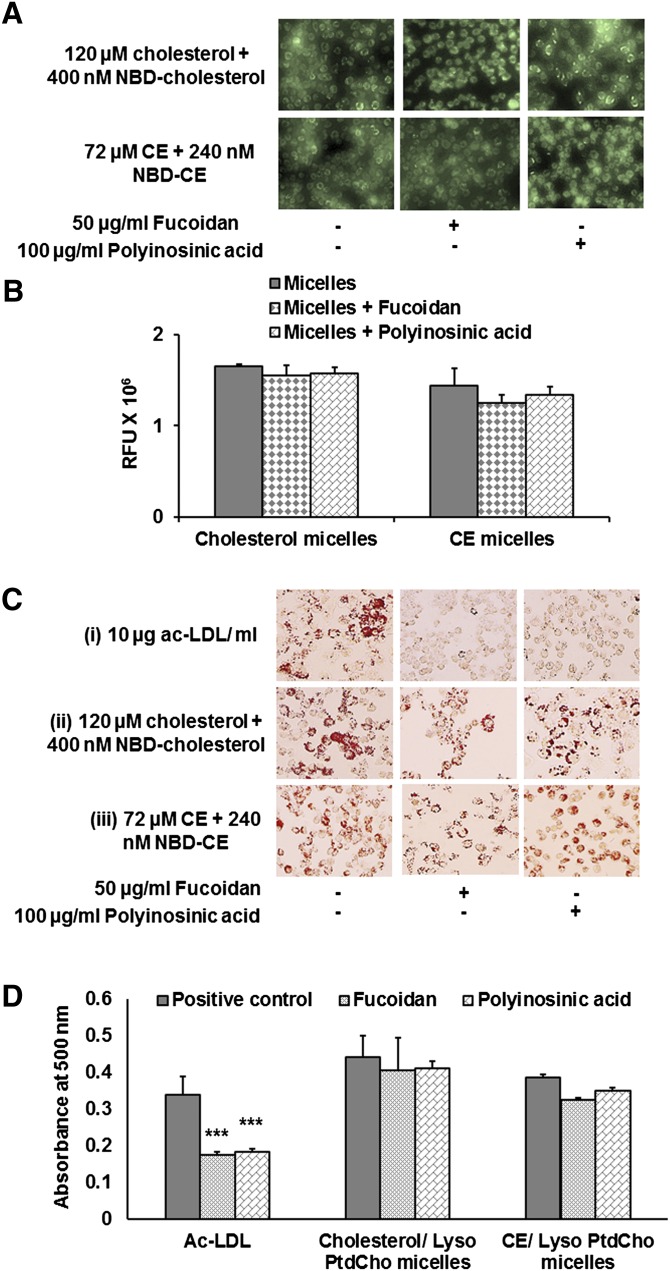
Mixed micelle-mediated foam cell formation is not affected by scavenger receptor binding ligands. (A) Macrophages were preincubated with either 50 µg/ml fucoidan or 100 µg/ml polyinosinic acid, followed by incubation with cholesterol or CE (unlabeled and fluorescently labeled)/lyso-PtdCho mixed micelles for 18 h. NBD-cholesterol or fluorescent CE accumulation within macrophages was observed in the cells preincubated with fucoidan and polyinosinic acid. (B) Measurement of fluorescence intensity in cell lysates from cells treated with fluorescently labeled cholesterol or CE with or without fucoidan or polyinosinic acid treatment. (C) Macrophages preincubated with fucoidan and polyinosinic acid show very little to no CE droplet accumulation after treatment with ac-LDL (i). CE droplet accumulation in macrophages preincubated with fucoidan and polyinosinic acid was observed after incubation with cholesterol (ii) or CE (iii)/lyso-PtdCho mixed micelles. (D) Oil Red O stain was eluted from the cells treated with ac-LDL and cholesterol or CE/lyso-PtdCho mixed micelles with or without the preincubation of fucoidan or polyinosinic acid, and then absorption of Oil Red O stain at 500 nm was measured. Values are expressed as mean ± SD (n ≥ 3). ***P < 0.005 (one-way ANOVA with Dunnett's multiple comparison test). Significant differences for fucoidan and polyinosinic treatment are compared with the respective positive control.

Fucoidan and polyinosinic acid are ligands for scavenger receptor A1 (SR-A1), and they reduce or inhibit the uptake of ac-LDL by macrophages (52, 53). However, fucoidan or polyinosinic acid did not inhibit the accumulation of fluorescent cholesterol or CE in RAW 264.7 macrophages (**Fig. 5A**, B). Fucoidan (50 µg/ml) and polyinosinic acid (100 µg/ml) were used separately to block ac-LDL-mediated foam cell formation. Oil Red O staining demonstrated that macrophages preincubated with fucoidan or polyinosinic acid accumulated very little to no CE droplets when treated with ac-LDL. But under the same conditions, macrophages incubated with cholesterol (or CE)/lyso-PtdCho mixed micelles accumulated plenty of CE droplets (Fig. 5C).

Quantification of CE droplet accumulation by Oil Red O stain elution further established this observation. While the optical density of stains eluted from cells treated with ac-LDL following preincubation with fucoidan or polyinosinic acid decreased by 2-fold from the positive control, the same preincubation had little or no effect on the CE droplet accumulation in cells treated with cholesterol or CE/lyso-PtdCho mixed micelles (Fig. 5D).

**Fig. 6.**
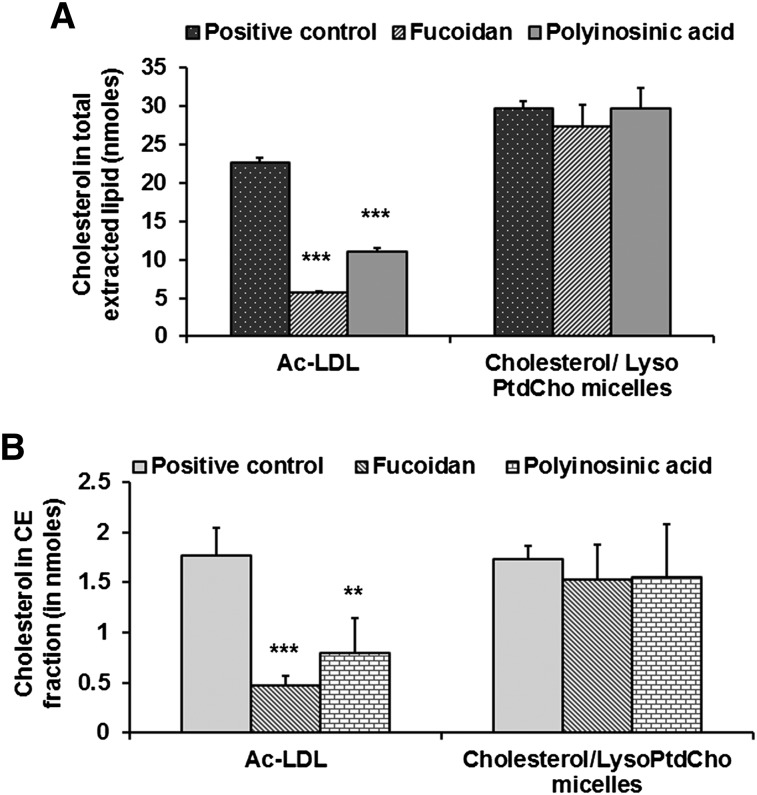
Incorporation of cholesterol is not inhibited by preincubation with fucoidan or polyinosinic acid. (A) RAW 264.7 macrophages were treated with ^3^H-cholesterol labeled ac-LDL or cholesterol, ^3^H-cholesterol/lyso-PtdCho mixed micelles with or without fucoidan and polyinosinic acid preincubation, and then incorporation of cholesterol in total extracted lipid was measured. (B) incorporation of cholesterol in CE fraction was measured from cells as treated above. Values are expressed as mean ± SD (n ≥ 3). **P < 0.01, ***P < 0.005 (one-way ANOVA with Dunnett's multiple comparison test). Significant differences for fucoidan and polyinosinic treatment are compared with the respective positive control.

The unique nature of foam cell development by mixed micelles was further established by using ^3^H-cholesterol-labeled ac-LDL and mixed micelles. Pretreatment of fucoidan and polyinosinic acid decreased the incorporation of cholesterol both in total extracted lipid and in a CE fraction by 4- and 2-fold, respectively, in the case of ac-LDL-mediated foam cell development. Fucoidan and polyinosinic acid did not have any effect on the incorporation of cholesterol in total extracted lipid or in the CE fraction (TLC plate, supplementary Fig. III) when foam cell development was mediated by cholesterol (unlabeled and ^3^H-cholesterol)/lyso-PtdCho mixed micelles (**Fig. 6A**, B).

### HDL causes cholesterol efflux from foam cells

**Fig. 7.**
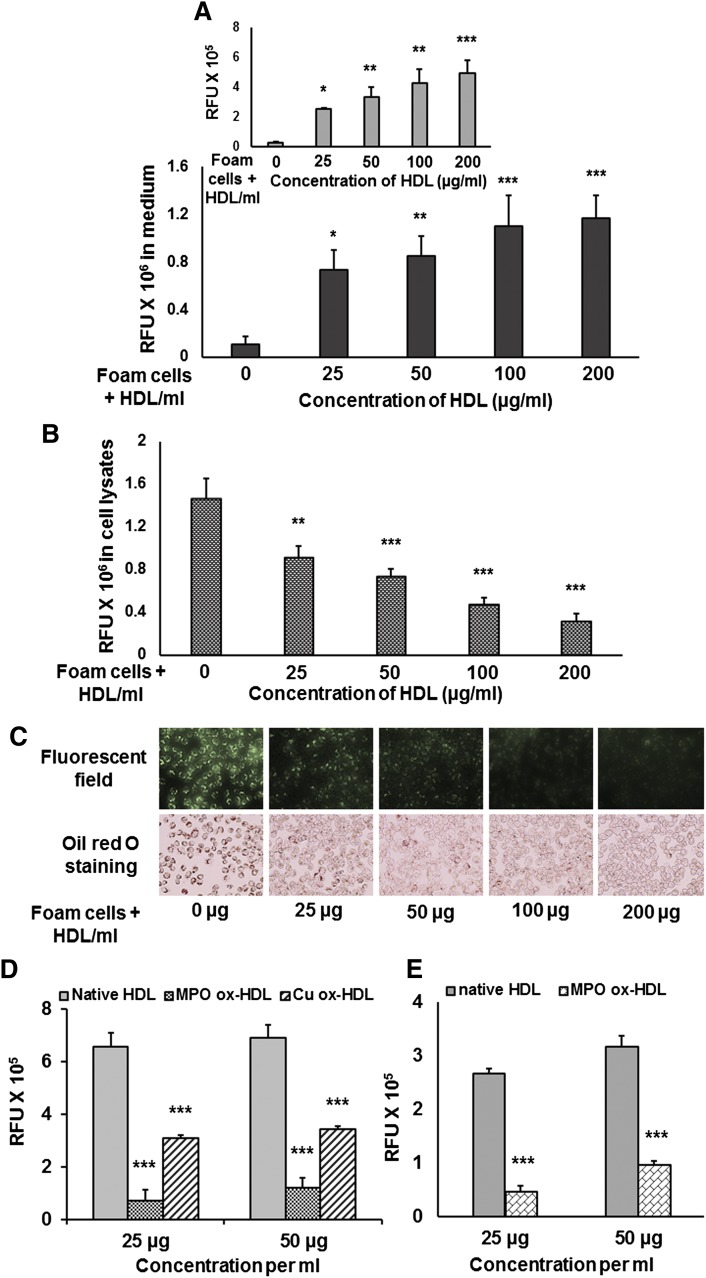
Cholesterol efflux from foam cells in presence of HDL. Foam cells were developed using cholesterol (unlabeled and NBD-cholesterol or ^3^H-cholesterol)/lyso-PtdCho mixed micelles. Foam cells were incubated with 0–200 µg/ml of HDL for 4 h. (A) Medium from NBD-cholesterol containing foam cells (RAW 264.7 macrophages) incubated with increasing concentrations of HDL showed concentration-dependent increase in fluorescence intensity. Insert shows mouse peritoneal macrophage-derived foam cells incubated with HDL demonstrate concentration-dependent NBD-cholesterol efflux in the medium. (B) Fluorescence intensity in the corresponding cell lysates decreases as the concentration of HDL increases. *P < 0.05; **P < 0.01, ***P < 0.005 (one-way ANOVA with Dunnett's multiple comparison test). (C) Fluorescence images and Oil Red O staining show reduction in CE droplet accumulations in cells after incubation with HDL. (D) Foam cells were incubated with 25 and 50 µg/ml of native HDL and MPO or Cu-mediated ox-HDL. Cholesterol efflux caused by native HDL was much higher when compared with the same concentration of ox-HDL. Values are expressed as mean ± SD (n ≥ 3). ***P < 0.005 (one-way ANOVA with Dunnett's multiple comparison test). Significant differences for ox-HDL treatments are compared with the corresponding concentration of native HDL. (E) Cholesterol efflux from peritoneal macrophage-derived foam cells incubated with 25 and 50 µg/ml of native HDL and MPO-mediated ox-HDL. Values are expressed as mean ± SD (n ≥ 3). ***P < 0.005 (two-tailed Student *t*-test).

Foam cells were developed by incubating RAW 264.7 macrophages and mouse peritoneal macrophages with cholesterol (unlabeled and NBD-cholesterol or ^3^H cholesterol)/lyso-PtdCho mixed micelles and used for the efflux study in presence of HDL. Foam cells were incubated with 0, 25, 50, 100, and 200 µg/ml of freshly prepared HDL. After 4 h, medium was collected from each well and RAW 264.7 macrophages were lysed to measure the presence of NBD-cholesterol in the medium and in the cell lysates, respectively. Fluorescence intensity in the medium collected from foam cells alone (no HDL) was in the background level and increased in a HDL concentration-dependent manner (**Fig. 7A**). A similar observation was made with respect to cholesterol efflux when mouse peritoneal macrophages were used (Fig. 7A, insert). Inversely, the fluorescence intensity in the lysate of foam cells without HDL incubation was the maximum. Fluorescence intensity in the lysate of foam cells decreased gradually as the concentration of HDL in the medium increased (Fig. 7B). Images were taken under fluorescence microscope after the incubation of foam cells with or without HDL. Reduction in fluorescence intensity indicated that less NBD-cholesterol remained inside the foam cells when they were incubated with increasing concentrations of HDL. Study of CE droplet accumulation by Oil Red O staining further confirmed our observations (Fig. 7C).

**Fig. 8.**
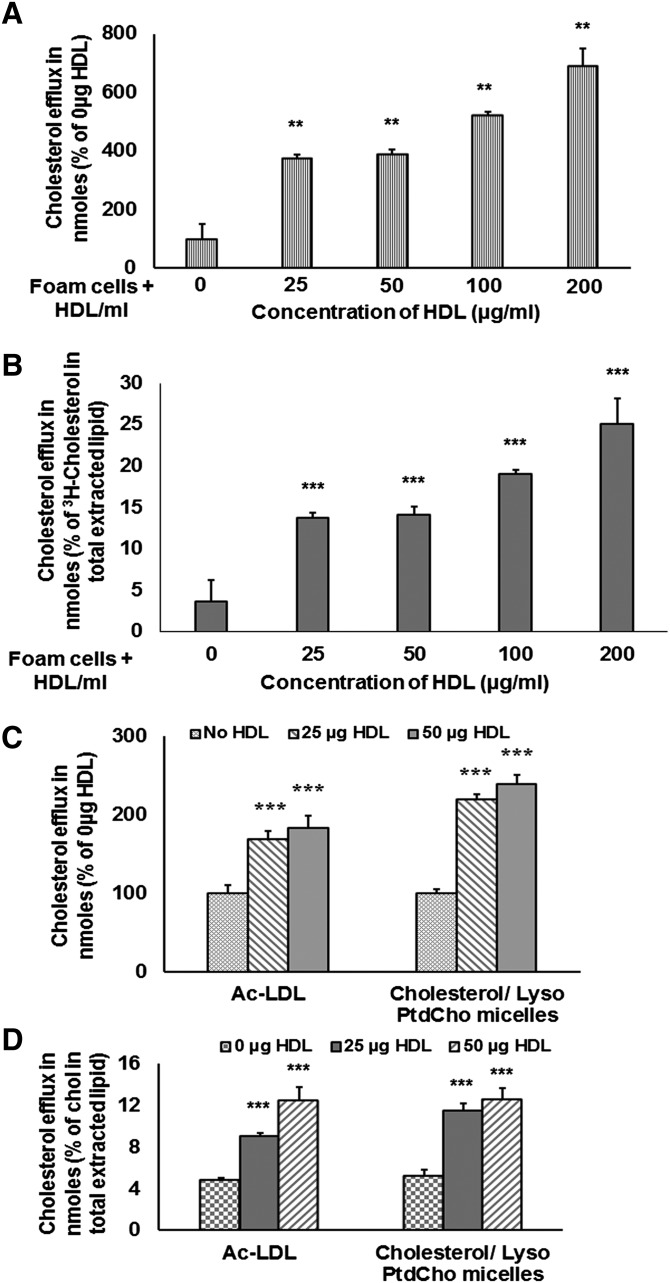
ac-LDL and mixed micelle-mediated foam cells demonstrate similar HDL-dependent cholesterol efflux. (A) foam cells developed by incubation with ^3^H-cholesterol containing mixed micelles showed concentration-dependent increase in cholesterol efflux when treated with increasing concentrations of HDL. (B) HDL-mediated cholesterol efflux was represented as percentage of cholesterol incorporated in total extracted lipid. (C) Foam cells were developed by incubating macrophages with ^3^H-cholesterol labeled ac-LDL or ^3^H-cholesterol containing mixed micelles, and HDL-mediated cholesterol efflux was studied using 25 and 50 µg/ml of native HDL. (D) HDL-mediated cholesterol efflux represented as percentage of cholesterol incorporated in total extracted lipid. Values are expressed as mean ± SD (n ≥ 3). **P < 0.01, ***P < 0.005 (one-way ANOVA with Dunnett's multiple comparison test).

These results were further validated in the efflux study using ^3^H-cholesterol. CPM value for ^3^H-cholesterol efflux was converted into nanomoles (**Fig. 8A**). Further analysis showed that 25–200 µg of native HDL caused cholesterol efflux ranging 10–25% of cholesterol present in total extracted lipid (Fig. 8B). Preliminary results indicated that most of the cholesterol in the medium was associated with intact, reisolated HDL (data not shown).

### HDL-mediated cholesterol efflux is quantitatively similar to foam cells developed by ac-LDL and mixed micelles

Foam cells developed by ^3^H-cholesterol labeled ac-LDL and cholesterol (unlabeled and ^3^H-cholesterol)/lyso-PtdCho mixed micelles were incubated with 0, 25, and 50 µg of native HDL for 4 h. Quantitatively, the cholesterol efflux by 25 and 50 µg of HDL from foam cells developed by mixed micelles were a little bit higher than that from ac-LDL-derived foam cells (Fig. 8C). However, when the percentage of cholesterol efflux was calculated with respect to cholesterol present in total extracted lipid, the efflux rate was very similar. Incubation with 25 and 50 µg of HDL caused 2- and 3-fold induction in cholesterol efflux from foam cells developed from either method of cholesterol loading (Fig. 8D).

### ox-HDL fails to cause cholesterol efflux

Cholesterol efflux from foam cells were compared in the presence of 25 µg and 50 µg native HDL and MPO and Cu-mediated ox-HDL. Cholesterol efflux was documented by measuring the fluorescence intensity in medium after incubation of foam cells with 25 µg and 50 µg native HDL and ox-HDL. Even though Cu-ox-HDL caused some cholesterol efflux, 25 µg and 50 µg native HDL induced much higher cholesterol efflux when compared with that of ox-HDL counterparts (Fig. 7D). As seen in Fig. 7D, MPO-mediated oxidation of HDL was more effective at inhibiting cholesterol efflux than was Cu-mediated oxidation. Cholesterol efflux in presence of ox-HDL was further confirmed by using NBD-cholesterol-loaded mouse peritoneal macrophages (Fig. 7E) and ^3^H-cholesterol-loaded foam cells (data not shown).

## DISCUSSION

In this study, we demonstrated for the first time that macrophage foam cells can be developed using micellerized cholesterol or CE. The foremost challenge to incorporate cholesterol in cells is obviously its high hydrophobicity. The amphiphilic property of lyso-PtdCho, which is also a natural detergent, was utilized to form mixed micelles and to solubilize cholesterol and CE (unlabeled and fluorescently labeled) in PBS. A previous study demonstrated that lyso-PtdCho could form a stable, soluble complex with equimolar cholesterol (54), and this technique has been modified successfully to solubilize β-carotene in aqueous solution (32). Augé et al. (32) further demonstrated that solubilized β-carotene could be utilized for cellular enrichment of β-carotene in vitro. In the present study, increasing amounts of cholesterol (unlabeled and NBD-cholesterol) were successfully solubilized in PBS by gradually raising the concentration of lyso-PtdCho in the mixed micelles. Solubility of NBD-cholesterol was demonstrated directly by measuring the fluorescence intensity of the aqueous solution. We validated the solubility of cholesterol in aqueous solution indirectly by reducing the solubility of NBD-cholesterol in a cholesterol concentration-dependent manner. As the concentration of cholesterol provided to make mixed micelles was gradually increased while keeping the concentration of NBD-cholesterol constant, the fluorescence intensity of the aqueous solution was decreased in a concentration-dependent manner. RAW 264.7 macrophages were loaded with NBD-cholesterol and unlabeled cholesterol in a concentration-dependent manner, and fluorescently labeled, cholesterol-enriched foam cells were developed. Foam cells were successfully generated by using mouse peritoneal macrophages as well. We also observed that mixed micelle mediated NBD-cholesterol uptake is a very rapid process; cells were enriched with NBD-cholesterol within 2 h continuing up to 24 h. Nonetheless, there was a sharp decline in the fluorescence intensity level at 48 h, which was due to detachment of many cells as a result of overconfluence.

Previous studies demonstrated that NBD-cholesterol has a higher aqueous solubility than cholesterol and might not truly represent the uptake and efflux of cholesterol (40). Higher solubility of NBD-cholesterol has been utilized for uptake studies by directly incubating cells with NBD-cholesterol in ethanol solution (29, 40). The influence of higher solubility of NBD-cholesterol in the results of cholesterol uptake and efflux studies was ruled out by using ^3^H-cholesterol, a more widely used cholesterol analog to repeat the key experiments. Recently, Khera et al. (28) and Li et al. (15) utilized macrophages labeled with free radioactive cholesterol for high-throughput cholesterol efflux studies. The enrichment of macrophages with free ^3^H-cholesterol or ^14^C-cholesterol was done by incubating cells with free radioactive cholesterol for 24 h. However, the goal of our present study was to develop foam cells enriched with cholesterol and CE, not just with fluorescent or radiolabeled cholesterol, by a novel, reproducible, quick technique. Therefore, lyso-PtdCho micelle-mediated cholesterol delivery was a more suitable approach to solubilize and to ensure the delivery of highly hydrophobic cholesterol and CE to the cells. In addition, we documented that the free cholesterol delivered by micelles gets esterified to CE and produces CE droplet-enriched foam cells. It is also to be noted that cellular cholesterol efflux is also accompanied by cellular phospholipid efflux. As demonstrated in our studies, lyso-PtdCho is efficiently converted to PtdCho and thus would provide the PtdCho needed for efficient efflux. It remains to be established whether the fluorescence/radioactivity of both cholesterol and PtdCho are associated with the HDL fraction in the medium. Our preliminary results (data not shown) suggest that most of the fluorescence in the medium is associated with isolated HDL after incubation with foam cells containing NBD-cholesterol.

Incorporation of ^14^C oleate was studied to ascertain that esterification of cholesterol into CE is indeed the source of lipid droplets seen inside the foam cells upon incubation with cholesterol/lyso-PtdCho micelles. Cells incubated with cholesterol/lyso-PtdCho micelles yielded the CE band comparable to one from CE/lyso-PtdCho micelle-treated cells upon TLC separation (supplementary Fig. II). Nonetheless, cholesterol/lyso-PtdCho micelle-treated cells showed very high ^14^C oleic acid incorporation, while CE/lyso-PtdCho micelle-treated cells had a 10-fold reduction in oleate incorporation. This demonstrated that cholesterol/lyso-PtdCho mixed micelles generated foam cells, which would be physiologically relevant, as cholesterol was esterified into CE and appeared as lipid droplets during Oil Red O staining. For all the experiments involving cholesterol (unlabeled and NBD or ^3^H-cholesterol)/lyso-PtdCho micelles, cells were incubated with 40 µM oleic acid along with the micelles. Oleic acid was supplied to facilitate the esterification of cholesterol into CE and has been used in many studies to follow ac-LDL uptake.

lyso-PtdCho is known to induce cell lysis due to its detergent-like properties, and it causes cell lysis by breaking down the intact cell membrane (54–56). It was observed in the current study that lyso-PtdCho alone is highly soluble in PBS, and it caused massive cell death. Cell lysis was confirmed by measuring secretion of LDH in medium upon incubation of cells with lyso-PtdCho micelles. However, cell morphology was not affected by the incubation with cholesterol/lyso-PtdCho mixed micelles, as is clearly demonstrated (Fig. 4A, iii). We also showed that red blood cells (RBC) were resistant to lysis when increasing concentrations of cholesterol were present along with lyso-PtdCho. This observation was not surprising, as the work of Rand et al. (54) explained that a stable complex between equimolar cholesterol and lyso-PtdCho causes a conformational change in micelle formation, leading to reduction in the destabilizing and lytic effects of lyso-PtdCho. The reduced lytic effects of cholesterol/lyso-PtdCho mixed micelles were clearly established by its reaction with RBCs. After incubation of RBC with lyso-PtdCho micelles for 15 min, lysed RBC released hemoglobin in the aqueous solution, while the same reaction with an equivalent amount of cholesterol/lyso-PtdCho mixed micelles showed very little or no cell lysis. Nonetheless, CE/lyso-PtdCho mixed micelles induced more cell lysis, causing increased secretion of LDH (data not shown) in the medium and hemoglobin in the aqueous solution when compared with that of cholesterol/lyso-PtdCho. One possible explanation could be that CE does not cause the conformational changes that cholesterol does during mixed micelle formation, and thus, CE is less effective in reducing the lytic effects of lyso-PtdCho. After this initial observation, reduced volume of CE/lyso-PtdCho mixed micelles was used to enrich macrophages with CE. Lytic effects of lyso-PtdCho micelles were clearly in effect even with the concentration as low as 10 µM of lyso-PtdCho, while presence of CE up to a concentration of 100 µM greatly reduced this lytic effect.

The advantage of using lyso-PtdCho as a membrane fusion agent to deliver cholesterol to cells is 2-fold: lyso-PtdCho is metabolized by cells into PtdCho (an acylation reaction) and into glycerophosphorylcholine (a transesterification reaction) (50). Previous studies demonstrated this conversion using various cell lines, including macrophages (51). The quick metabolism of lyso-PtdCho by cells ensures successful cargo delivery to the cells without influencing RCT. We observed that more than 75% of lyso-PtdCho delivered as mixed micelles was metabolized into PtdCho within 2 h of incubation of cells with cholesterol/lyso-PtdCho (unlabeled and ^14^C-tagged) mixed micelles.

We further established that micellerized CE uptake is uniquely distinguished from ac-LDL uptake. SR-A1 is actively involved in ac-LDL binding and ac-LDL mediated uptake of cholesterol (52). We used fucoidan and polyinosinic acid, known ligands for SR-A1 (53), to inhibit the binding of ac-LDL to SR-A1. As expected, fucoidan and polyinosinic acid prevented the ac-LDL-mediated foam cell formation and greatly inhibited the incorporation of ^3^H-cholesterol in total extracted lipid and in the CE fraction of lipids in ac-LDL-treated cells. However, SR-A1 ligands had no effect on cholesterol or CE/lyso-PtdCho mixed micelle-mediated cholesterol delivery, and the accumulation of NBD-cholesterol remained unaffected. Additionally, fucoidan and polyinosinic acid had no inhibitory effect on the incorporation of ^3^H-cholesterol in total extracted lipid or on the CE fraction of lipids when macrophages were incubated with cholesterol/lyso-PtdCho mixed micelles. Thus the micelle-mediated cholesterol or CE delivery utilizes the membrane fusion property of lyso-PtdCho, making this foam cell development technique novel and unique. Furthermore, the universality of micelle-mediated delivery was established by developing cholesterol-enriched hepatocytes, HepG2 cells (data not shown).

Currently the development of foam cells used to study RCT typically involves isolation and acetylation of LDL, followed by incorporation of radiolabeled cholesterol. Isolation of LDL is a tedious process, and LDL is prone to be oxidized automatically during the isolation process (1). Isolation and acetylation of LDL can vary qualitatively from one preparation to another, resulting in the induction of inflammatory responses in animal models. Most importantly, ac-LDL preparations are not stable and cannot be stored for a long time. In our present study, we established that cholesterol or CE/lyso-PtdCho mixed micelle preparation is functionally stable at 4°C and −20°C up to one month and that mixed micelle preparation stored at −20°C is stable for even longer time (data not shown). Furthermore, mixed micelle preparation stored at −20°C for one month was used to develop macrophage foam cells, which were qualitatively similar to those developed with freshly prepared mixed micelles. Quantitative analysis of foam cells developed with mixed micelles stored at 4°C and −20°C up to one month revealed that storage time did not affect the accumulation of NBD-cholesterol or CE droplets or the incorporation of ^3^H-cholesterol in total extracted lipid or the CE fraction of the lipid. Extraction of lipids from the mixed micelle preparation stored at 4°C and −20°C for several weeks followed by analysis from TLC separation showed no decrease in lyso-PtdCho and cholesterol content (data not shown).

We also observed that mixed micelle-mediated cholesterol loading was highly reproducible and the amount of cholesterol incorporated in the total extracted lipid always ranged 25–27 nmol when cells were incubated with cholesterol (unlabeled and ^3^H-cholesterol)/lyso-PtdCho mixed micelles (data not shown). The same treatment resulted in the incorporation of 1.5–1.8 nmol of cholesterol in CE fraction of the lipid when the experiment was repeated five times or more (data not shown).

Foam cells developed by using cholesterol/lyso-PtdCho mixed micelles were efficiently used to study cholesterol efflux. Increased concentration of HDL caused a concentration-dependent cholesterol efflux from the foam cells and was quantified by measuring the amount of NBD-cholesterol in the medium. For the efflux study, foam cells were also developed using cholesterol (unlabeled and ^3^H-cholesterol)/lyso-PtdCho mixed micelles, and concentration-dependent efflux of ^3^H-cholesterol confirmed our observations using NBD-cholesterol. HDL-mediated cholesterol efflux from foam cells developed by mixed micelles was quantitatively very similar to foam cells developed by ac-LDL. Various studies have suggested that oxidation of HDL leads to loss of its antiatherogenic property. To determine whether the micelle-derived foam cells could be utilized to differentiate between native, functional HDL and oxidized, dysfunctional HDL, we used equal concentrations of native and ox-HDL separately. As we expected, ox-HDL failed to induce any significant cholesterol efflux from the foam cells. Therefore, we conclude that the foam cells developed by using cholesterol/lyso-PtdCho mixed micelles potentially can be used to study RCT in subjects with or without the background of cardiovascular disease.

Development of foam cells loaded with fluorescently labeled cholesterol via mixed micelles mediated technique would be less time consuming and highly reproducible. Furthermore, these foam cells could be utilized to screen a large number of human plasma/HDL samples to determine the efficacy of cholesterol efflux from foam cells or RCT.

## Supplementary Material

Supplemental Data
