# Supplementary material for: Novel technique for generating macrophage foam cells for in vitro reverse cholesterol transport studies
Source: J Lipid Res. 2013 Dec;54(12):3358–72. doi: 10.1194/jlr.M041327 (PMC3826683; doi:10.1194/jlr.M041327)
Supplement: Supplemental Data [file supp_54_12_3358__index.html]

Novel technique for generating macrophage foam cells for in vitro reverse cholesterol transport studies — Novel technique for generating macrophage foam cells for in vitro reverse cholesterol transport studies — Supplemental Data 

# Novel technique for generating macrophage foam cells for in vitro reverse cholesterol transport studies

## Supplemental Data

**Files in this Data Supplement:**

- Supplementary figure 1 - TLC of extracted lipid- stability of micelles
- Supplementary figure 2 - TLC of extracted lipid- oleate incorporation
- Supplementary figure 3 - TLC of extracted lipid- Ac-LDL and micelle loading
