# Supplementary material for: Novel technique for generating macrophage foam cells for in vitro reverse cholesterol transport studies
Source: J Lipid Res. 2013 Dec;54(12):3358–72. doi: 10.1194/jlr.M041327 (PMC3826683; doi:10.1194/jlr.M041327)
Supplement: Supplemental Data [file supp_M041327_jlr.M041327-1.pdf]

Supplementary figure 1.

Extracted lipid was separated on TLC plate and TLC plate exposed to Iodine vapor. Cholesterol (unlabeled and ^{3}H-cholesterol)/ Lyso PtdCho mixed micelles stored at room temperature, 4° C and -20° C for Week 0, Week 1, Week 2 and Week 4 were used to incubate RAW 264.7 macrophages for 18 hours. Extracted lipid was used to run TLC with cholesterol, cholesteryl ester and Lyso PtdCho standards and one of the representative TLC plate was shown here. TLC plate was cut into eight sections for each sample and radioactivity measurement was taken as shown in the table.

Supplementary figure 1.

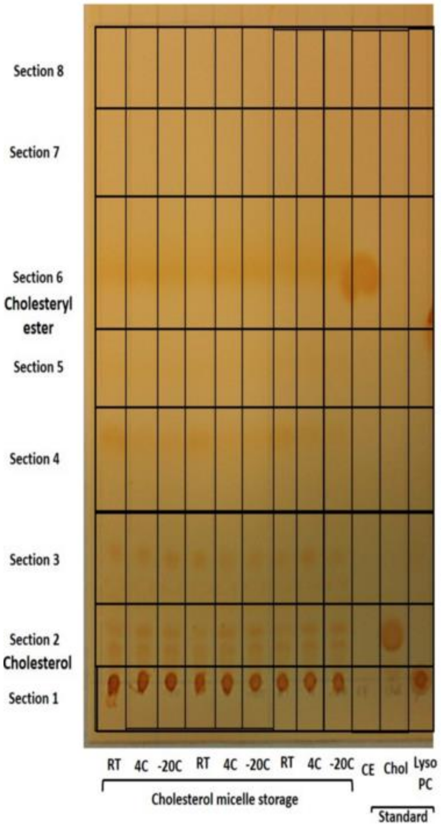

	Week 0	Week 1			Week 2			Week 4		
Cholesterol / Lyso PtdCho micelles	Week 0	RT	4C	-20C	RT	4C	-20C	RT	4C	-20C
Section 8	-6.66667	-7.77778	-6.44444	-7.88889	-1.11111	-3.94444	-1.61111	3.5	2	5
Section 7	-8.77778	-7.55556	-7.88889	-8.22222	-2.11111	-4.44444	2.888889	4.833333	2.375	4.25
Section 6	1732.889	936.3333	1328.667	1372.111	822.3889	1253.889	1362.806	530.3333	1250.75	1391
Section 5	49.66667	108.1111	173.8889	167.5556	226.5556	328.8889	308.8889	94.16667	202.25	182.875
Section 4	27.33333	23.22222	41.55556	27.66667	51.88889	69.55556	70.38889	62.83333	144	115.375
Section 3	94.11111	82.66667	95.66667	98.88889	137.7222	177.7222	184.5556	92.33333	150.375	129
Section 2	13603.56	10922.33	11690.89	12384.44	8995.222	10540.78	11081.56	9354.833	9754.125	10908
Section 1	878.3333	850.1111	870.8889	885.4444	776.8889	938.8889	1041.222	818.1667	862.875	858.5
