# Supplementary material for: Novel technique for generating macrophage foam cells for in vitro reverse cholesterol transport studies
Source: J Lipid Res. 2013 Dec;54(12):3358–72. doi: 10.1194/jlr.M041327 (PMC3826683; doi:10.1194/jlr.M041327)
Supplement: Supplemental Data [file supp_M041327_jlr.M041327-2.pdf]

Supplementary figure 2.

Extracted lipid was separated on TLC plate and TLC plate exposed to autoradiography imager followed be exposure to Iodine vapor. Cholesterol/ Lyso PtdCho and CE/ Lyso PtdCho mixed micelles along with ^{14}C-Oleic acid were used to incubate RAW 264.7 macrophages for 18 hours. Extracted lipid was used to run TLC with cholesterol, cholesteryl ester, oleic acid and Lyso PtdCho standards. TLC plate was cut into eight sections for each sample and radioactivity measurement was taken as shown in the table.

Supplementary figure 2.

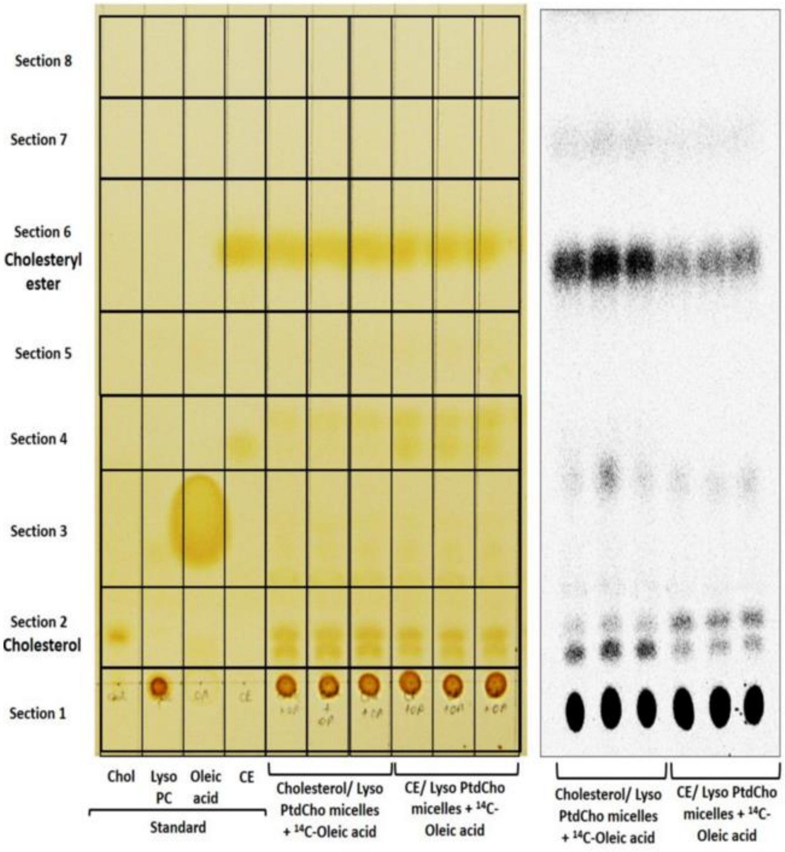

	Cholesterol/ Lyso PtdCho micelles + ^{14}C-Oleic acid	CE/ Lyso PtdCho micelles + ^{14}C-Oleic acid
Section 8	5	2.666667
Section 7	7	5.333333
Section 6	27462.5	1063.667
Section 5	1544.333	778.5
Section 4	11480.5	5271.833
Section 3	2442.667	984.6667
Section 2	3936	3526.333
Section 1	140311.5	95780.17
