# Supplementary material for: Novel technique for generating macrophage foam cells for in vitro reverse cholesterol transport studies
Source: J Lipid Res. 2013 Dec;54(12):3358–72. doi: 10.1194/jlr.M041327 (PMC3826683; doi:10.1194/jlr.M041327)
Supplement: Supplemental Data [file supp_M041327_jlr.M041327-3.pdf]

Supplementary figure 3.

Extracted lipid was separated on TLC plate and TLC plate exposed to Iodine vapor. ^{3}H-cholesterol tagged ac-LDL and cholesterol (unlabeled and ^{3}H-cholesterol)/ Lyso PtdCho mixed were used to incubate RAW 264.7 macrophages with or without fucoidan or polyinosinic acid for 18 hours. Extracted lipid was used to run TLC with cholesterol and cholesteryl ester standards. TLC plate was cut into eight sections for each sample and radioactivity measurement was taken as shown in the table.

Supplementary figure 3.

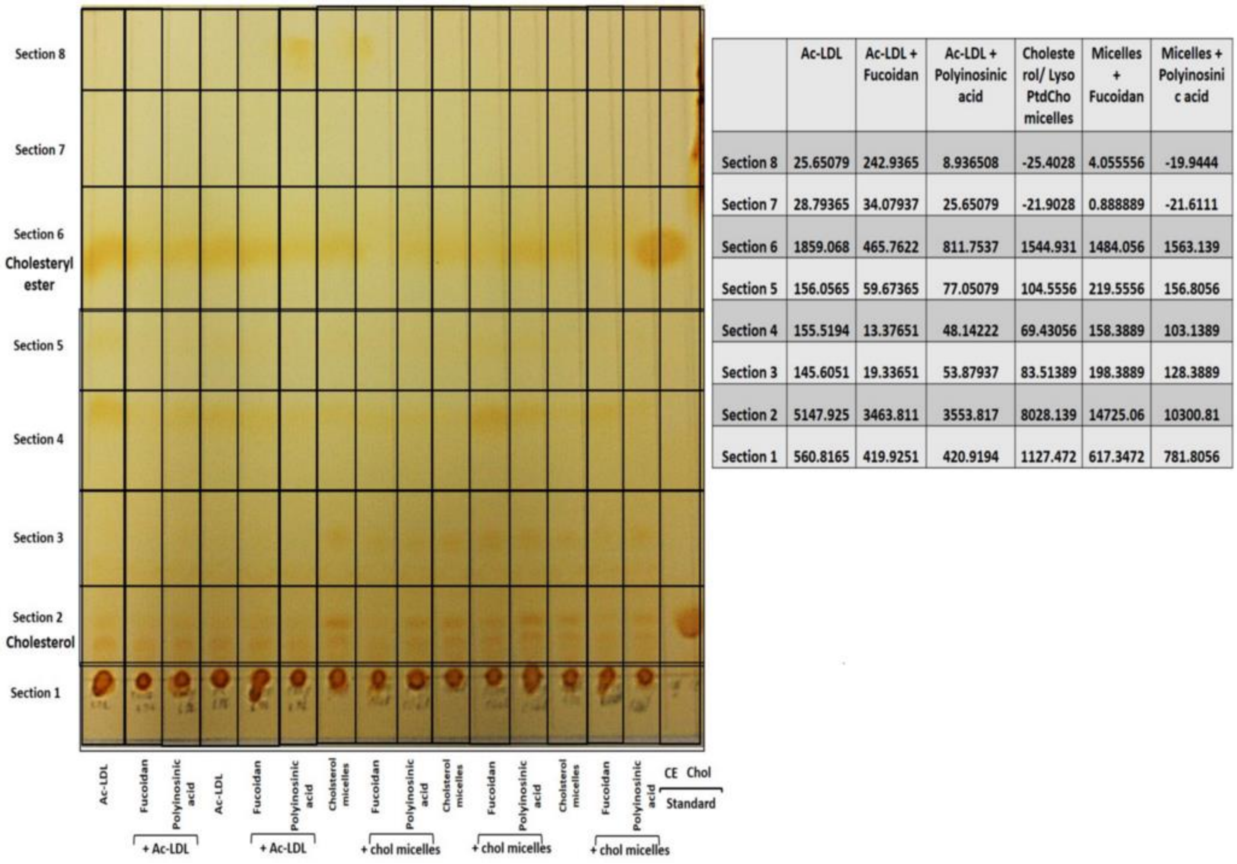

	Ac-LDL	Ac-LDL + Fucoidan	Ac-LDL + Polyinosinic acid	Cholesterol/ Lyso PtdCho micelles	Micelles + Fucoidan	Micelles + Polyinosinic acid
Section 8	25.65079	242.9365	8.936508	-25.4028	4.055556	-19.9444
Section 7	28.79365	34.07937	25.65079	-21.9028	0.888889	-21.6111
Section 6	1859.068	465.7622	811.7537	1544.931	1484.056	1563.139
Section 5	156.0565	59.67365	77.05079	104.5556	219.5556	156.8056
Section 4	155.5194	13.37651	48.14222	69.43056	158.3889	103.1389
Section 3	145.6051	19.33651	53.87937	83.51389	198.3889	128.3889
Section 2	5147.925	3463.811	3553.817	8028.139	14725.06	10300.81
Section 1	560.8165	419.9251	420.9194	1127.472	617.3472	781.8056
